# “It Is a Very Emotional Topic for Me”—Managing Breastfeeding Problems among German Mothers: A Qualitative Approach

**DOI:** 10.3390/healthcare9101352

**Published:** 2021-10-11

**Authors:** Mariz Spannhake, Charlotte Jansen, Tatiana Görig, Katharina Diehl

**Affiliations:** Medical Faculty Mannheim, Mannheim Institute of Public Health, Social and Preventive Medicine, Heidelberg University, Ludolf-Krehl-Straße 7–11, 68167 Mannheim, Germany; mariz_spannhake@gmx.de (M.S.); charlotte.jansen@medma.uni-heidelberg.de (C.J.); Tatiana.Goerig@medma.uni-heidelberg.de (T.G.)

**Keywords:** breastfeeding, difficulties in breastfeeding, breast pump, stress, children, mothers, qualitative study, Germany

## Abstract

Breastfeeding is associated with positive health outcomes for both child and mother. Nevertheless, some women experience breastfeeding problems which commonly lead to early cessation, or not starting breastfeeding at all. Our aim was to explore how women that have difficulties in breastfeeding perceive their situation and how they actively manage it. We conducted semi-structured interviews with 15 mothers living in Germany who had experienced breastfeeding problems. The interviews were audio-recorded, transcribed verbatim, and analyzed using qualitative content analysis. Breastfeeding problems occurred due to different reasons and had a huge impact, as evidenced in the four main themes of the findings: individual situation, managing the situation, perceived consequences for relations, feelings, and potential future pregnancies, and perceived health consequences for the mother. They frequently experienced negative emotions, including psychological distress and mental health problems, with perceived negative consequences for the mother–infant-bonding. Trying to actively manage the situation and availability of social support seemed to have a relieving effect, whereas confrontation and lack of understanding worsened the situation. Breastfeeding problems and the inability to breastfeed can have a great influence on maternal well-being. These can affect different aspects of a mother’s life, including the attachment to the child. Providing support for actively managing the situation and supporting the exchange of experience between mothers who perceive breastfeeding problems may help mothers to better deal with their situation. Our findings may help health professionals to understand what these mothers feel and how they can support these women in a sensitive way.

## 1. Introduction

Breastfeeding is associated with many positive health outcomes for a child and the mother [1,2,3,4,5,6]. Besides reducing childhood mortality [7,8], it has several positive long-term effects that can even last into the child’s adulthood, for example, in reducing the risk of type 2 diabetes [9,10] and obesity [9,11]. The direct benefits for the mother include a decreased risk for gynecological tumors, for example, breast cancer [12] and ovarian cancer [13]. Based on these breastfeeding benefits and in ensuring a child’s survival, the World Health Organization (WHO) recommends the exclusive breastfeeding of a newborn child for the first six months of life [14].

Unfortunately, the global breastfeeding rate has not reached the recommended duration, nor does every woman actually start breastfeeding in the first place. In high-income countries, the prevalence of mothers ever breastfeeding is much lower compared to countries with lower income [8]. While 87% of mothers in Germany start to breastfeed after birth, only 46% of babies are breastfed exclusively for at least four months [15]. This means that 13% of mothers did not start breastfeeding at all, while others would stop breastfeeding relatively early. The main reasons for weaning in the first six months are too little breast milk, breast inflammation, and other health problems [15,16]. Rated as less important reasons are social ones, for instance, resuming work after maternity leave. Besides that, studies from other countries show that many women face problems when starting breastfeeding, resulting in early cessation of breastfeeding [17,18,19]. However, little is known about how women who are unable to breastfeed or are faced with breastfeeding problems perceive this situation.

Only a few studies have focused on mothers having problems with breastfeeding. According to a Chilean qualitative study, the affected mothers suffered from frustration and despair [20]. Another study indicated that mothers who could not breastfeed their children as planned had a higher risk of developing postpartum depression [21].

The current state of research shows that the situation of women who are unable to breastfeed has been understudied. Little is known about how these mothers perceive their situation, what feelings and health consequences they experience, and how they manage these. Therefore, our aim was to explore this topic through the use of semi-structured interviews following a phenomenological research design in a group of women who had breastfeeding problems or could not breastfeed naturally. Our research questions were as follows: (1) How did women who were unable to breastfeed naturally or had to stop breastfeeding early perceive their situation?; (2) How did these women try to actively manage their situation?; (3) How did these women perceive their situation regarding personal relationships, own feelings, and consequences for potential future pregnancies?; (4) Which health consequences did these women experience related to their breastfeeding problems? Our findings will help to give some idea on how these women perceive their situation and what potential support could be provided.

## 2. Materials and Methods

A qualitative study following a phenomenological design was conducted among mothers who had breastfeeding problems or had to stop natural breastfeeding early. The focus of this manuscript is on the following four main themes and their subthemes: (1) individual situation regarding breastfeeding problems, (2) managing the situation, (3) perceived consequences for relations, feelings, and potential future pregnancies, and the (4) perceived health consequences for the mothers. The participants were recruited as a convenient sample via snowballing, on recommendation from midwives and private contacts throughout Germany. The inclusion criteria were: motherhood, an early unwanted stop in breastfeeding with at least the first child, or the inability to breastfeed at least the first child, and sufficient German language skills to participate in the interview. No further inclusion or exclusion criteria were applied. Theoretical saturation was experienced at a saturation point of approximately 12 interviews, which means that the gain of new information would decrease with the addition of interviews. Therefore, recruitment was stopped after 15 interviews. Semi-structured, face-to-face, and telephone interviews with open-ended questions were used in the data collection.

The interviews were conducted between 6 June 2019 and 22 October 2019 by M.S. (female medical student who is also a trained nurse), which lasted for 52 min on average (min: 27 min, max: 90 min). Prior to the interviews, M.S. was extensively trained by the last author (K.D.), who is an experienced interviewer. For face-to-face interviews, the participants suggested the locations where they would feel comfortable to be interviewed and to respond. For interviews conducted via telephone, the participants chose a private place where they would not be disturbed. No third parties were present during the interviews, except in some cases, the participant’s own baby or toddler. All interviews were audio-recorded and transcribed verbatim. Prior to the interviews, the participants were informed about the study’s procedure and data protection. All participants provided written (personal interviews) or verbal (telephone interviews) consent to participate in the study. They received a €20 gift voucher each as a reimbursement for their time. Approval was obtained from the Ethics Committee II of the Medical Faculty Mannheim, Heidelberg University on 26 March 2019 (number 2019-640N).

We used qualitative content analysis following Mayring (2010) to analyze the transcripts. We systemized the transcribed interviews by identifying the categories and subcategories (i.e., the common themes within the interviews). Based on the semi-structured interview guide, we developed the initial set of main codes that was further complemented during the coding process. We coded the data using the MAXQDA program (VERBI GmbH, Berlin, Germany, Version 20.3.0). The interviews were independently coded by two researchers (MS and CJ). Disagreements were discussed by the two coders and resolved through a consensus in each case.

## 3. Results

Altogether, 15 mothers participated in the study. On average, they were 32.6 years old (SD = 5.3, min = 22, max = 41). The majority of them had one child (73%), three women had two children (20%), and one woman had three children (7%). On average, the firstborn child was 32.9 months old (SD = 19.68, min = 6, max = 76). On average, the women were 29.7 years old (SD = 5.3, min = 18, max = 37) when they gave birth to their first child. All women had breastfeeding problems with the first child. Two mothers had breastfeeding problems with the second child too (M05, M06), while two mothers were able to breastfeed their second child (M02, M14). All women were born in Germany and were native German speakers (for additional sociodemographic details, refer to Table 1).

Four main themes could be extracted from the interviews (Table 2): (1) individual situation regarding breastfeeding (see Section 3.1), (2) managing the situation (see Section 3.2), (3) perceived consequences for relations, feelings, and potential future pregnancies (see Section 3.3), and (4) perceived health consequences for the mother (see Section 3.4).

### 3.1. Individual Situation Regarding Breastfeeding

All women had problems with breastfeeding. Some were unable to breastfeed at all, while others tried it as long as they could. These attempts took different periods, and most of the women suffered because of the situation. For instance, M15 reported that it was *“always a struggle”*, and M10 told us that she *“cried her eyes out”*, because *“[name of the baby] screamed at my breasts and didn’t get any milk out by her own”*. The interviews showed that the topic was very emotional for many of the women since they would start to cry, or strive to refrain from crying throughout the session.

The presumed main reasons for not being able to breastfeed are manifold (Online Supplement Materials, Table S1). Most of the reported reasons were the baby itself (M01, M02, M07, M09, M10, M11, M12, M14, M15). The women often named a general weakness or reluctance of the infant to suck on the breast, for example, M10, who said: *“It was up to [name of the child]. She just couldn’t suck properly.”* M03, among others, blamed an early start of (additional) bottle-feeding (M02, M03, M05, M06, M07, M08, M13, M14) for the breastfeeding problems: *“It came out of the bottle much faster, it was always readily available, even in the amount he wanted.”* Another perceived reason was an insufficient amount of breast milk (M01, M03, M05, M06, M12). M06 stated that *“nothing came out”* of the breast. *“Milliliters only came. Ten to twelve with great difficulty, and that was too little. Of this, you cannot feed a child”* (M06). Five participants mentioned that the inability to breastfeed was also related to their own mental condition (M04, M05, M06, M11, M12). For instance, M11 told the interviewer: *“Because of the psychological pressure that you put on yourself, nothing (i.e., breast milk) came out.”* Further reasons, with each named four times, were problems with correct latching (e.g., M07: *“I think the biggest problem was that I could never really latch him correctly.”*), painful breast (e.g., M08: *“And the problem was at that point in time, my breasts/my nipples were already totally inflamed […].”*), a delivery through Caesarean section (e.g., M13: *“[…] and then when we were at home, the midwife said to me: “Yes, it often does not work [i.e., breastfeeding] after a caesarean section,” […].”*), and a separation of mother and child after birth due to different reasons (e.g., M12: *“[…] because I saw him for two minutes after giving birth, and then he was on the intensive child care unit and I was upstairs [on another unit].”*). Three women wondered whether it could be due to potential genetic reasons (M01, M05, M13). M01 said: *“So my mother says she couldn’t breastfeed me either. I was bottle-fed from the beginning when I was little. Maybe it’s just in the family.”* Health problems of the mother were also named as a potential reason (M05, M06, M13). For example, M05 told us: *“You have to squeeze the front of the breast so that the child can dock properly. But that was not possible with me.”*

### 3.2. Managing the Situation

This paragraph describes our findings on how the women managed their situation regarding feeding their babies (Section 3.2.1) and how cognitive–emotional responses related to managing the situation looked like (Section 3.2.2).

#### 3.2.1. Active Managing Regarding Feeding of the Baby

To cope with their situation, three mothers searched on the Internet and read books to get assistance (M03, M07, M08). Three mothers sought help from health professionals besides their midwives (M03, M07, M09). Two mothers reported that talking to other, more independent people was helpful (M01, M05). M01 told the interviewer about consolation by a distant relative, saying: *“She told me “It didn’t work for [my cousin] either. It’s not bad at all! You weren’t breastfed either, you grew up” […] and it’s true, we grew up, too!”*

Fourteen mothers tried nipple shields to facilitate nursing for the baby. However, it did not help in most of the cases, or only for a short time. All 15 mothers tried using breast pumps. Most mothers were not very successful (M01, M03, M05, M06, M08, M10, M11, M12, M15) and used breast pumps only for a short while. Many women did not have enough breast milk, for instance, M01 stating: *“it just didn’t come out a lot”*, or M03 saying: “*you sit there with the breast pump and look at every milliliter that drips […] in slow motion, you have 20 mL at some point after pumping and you think, “That’s just not possible.””*. Others had pain while using the breast pump, for example, M05 who reported that it took her two hours to pump the milk, and that her daughter got: *“strawberry milk, because my nipple bled”*. Using a breast pump was seen as stressful and exhausting by some of the women [M01, M03, M06, M08, M10, M12]. M10 concluded: *“It’s not nice. You really only do this so that the child gets something [i.e., breast milk]. And that it gets the best. It is actually nothing more and nothing less. It is like a cow.”*

Mothers who were more successful (M02, M04, M07, M09, M13, M14) did not perceive using the breast pump as such a heavy load and used it for a longer time. For some of these, it was a relief that “at least” pumping breast milk worked out. M02 reported that it was a *“really good feeling, that I had enough breast milk to pump it. That helped me a lot. I think, if I could not have pumped breast milk, it [the situation] would have been even more difficult for me to bear.”* As a result, they pumped as much milk as possible (e.g., M04: *“I pumped like a maniac.”*).

Resources that were used by these mothers to relieve breast pain were balm, quark poultice, cool pads, and teabags. Massages, special nursing teas, nutritional supplements, globules, plant products (e.g., fenugreek), and malt beer were used to boost the production of breast milk. Thirteen mothers used supplementary formula (at least for some period of time) because they were unable to pump breast milk or did not have enough breast milk (e.g., M12: *“I had to feed baby formula regardless”*). The step taken in using supplementary formula was also, in some cases, related to the development of the child (e.g., M11: *“Otherwise he would have been undernourished”*). Although using formula was associated with less stress (e.g., M09: *“[…] we were both more relaxed with the bottle”*), the step to the exclusive use of formula was not easy for all women (e.g., M01: *“Well, my husband said: “Then it just doesn’t work and then we just get formula!” So he was completely pragmatic. And it was hard for me.”*).

#### 3.2.2. Cognitive–Emotional Responses Related to Managing the Situation

Managing the situation was associated with cognitive and emotional responses of the women (Supplementary Materials: Table S2). All of them described more than one response. These included self-doubts and feelings of failure when breastfeeding did not work (M01, M02, M03, M04, M07, M09, M11, M13, M14, M15). M01 revealed: *“It takes a lot out of you when you think: “Oh, I’m the biggest loser because I just can’t breastfeed, yes! […].”* A lot of participants also felt burdened with an additional amount of work due to the use of the breast pump or formula (M02, M03, M05, M09, M10, M12, M13, M14, M15). For example, M09 told us: *“So we always had a huge bag full of things with us when we were traveling, even if we only went into town,”* and M12 found pumping breast milk *“a bit annoying and exhausting”*. Eight women also mentioned that they experienced stress and pressure (M03, M06, M08, M09, M10, M11, M13, M15). M06 explained that *“[…] you put yourself under a lot of stress because you wanted it [i.e., breastfeeding] to work out”*. Guilt feelings towards the child were experienced by seven women because they could not breastfeed (e.g., M07: *“[…] yes, the guilty conscience was, I guess, the biggest problem.*”), and even concerns about the child and its health arose (M03, M05, M07, M08, M10, M13). M08 described her thoughts as follows: *“I’ve always been more nervous because this baby just didn’t drink.”* Several participants also felt disappointment because of the unaccomplished expectations (M01, M02, M03, M04, M11, M15). For instance, M04 said: *“Yes, everything was somehow not as I had imagined.”* Mental overload, as well as misery were each described by five women. M05, for example, was overwhelmed with breastfeeding after birth: *“No one has ever looked after me or my daughter [in the delivery room after birth]. […] But in the end I was left alone. […] And nobody helped me that my child was fed.”*, and M15 experienced deep sadness due to the situation: *“I was really sad, that it [i.e., breastfeeding] did not work out.”* Frustration and rage were also named as cognitive–emotional responses (M06, M08, M09). M08 conceded: *“I was extremely annoyed because somehow it [i.e., breastfeeding] didn’t work at all.”* Three participants also mentioned that they were rationalizing to overcome the situation (e.g., M14: *“[…] I am a very pragmatic person and when it [i.e., breastfeeding] doesn’t work, then it does not work.”*).

### 3.3. Perceived Consequences for Relations, Feelings, and Potential Future Pregnancies

The following section describes our findings on how women perceived the relationship to their baby (Section 3.3.1), their partner (Section 3.3.2), and related persons (Section 3.3.3). In addition, the experience of mothers who were able to breastfeed is explored (Section 3.3.4). Finally, feelings of the women after having stopped breastfeeding (Section 3.3.5) and perceived consequences for potential future pregnancies are presented (Section 3.3.6).

#### 3.3.1. Interaction with the Child

Seven mothers reported changes in their interactions with their children. These changes included physical and/or emotional distancing (M04, M05, M08, M14, M15), being worried (M04), and intensified search for closeness (M07, M12, M15).

Distancing from the child was reflected, for instance, in the following quotation by M15: *“Yes, I would say that breastfeeding has our relationship/so it suffered a lot. It was like I said, when he woke up and I knew he was hungry, it was like that for me again “Oh no, he wants to be breastfed again and then we’ll sit for one and a half hour.” And I was crying again because I assumed that it would not work. […] Yes and I gave him [i.e., the baby] a lot to my husband. So at the beginning, according the motto “take him, take him, I can’t carry him and I already have so much” and that didn’t do us any good. […] But it got better and better when I stopped breastfeeding.”*

The intensified search for closeness with the child was reported, for example, by M07: “*I just tried to compensate a lot with him, also with the closeness. I hardly wanted to give him to anyone else, simply because this breastfeeding relationship did not exist.”* The perceived lack of bonding between a mother and her child was also emphasized by M15: *“[…] It is said that this [i.e., breastfeeding] builds the bond with the child and I was very afraid that we would not be able to do it. But in retrospect I can say that mothers who do not breastfeed get as much attachment to their child as mothers who breastfeed. It’s just like that. We did it in other ways. […] Maybe breastfeeding is a bit easier […], but ultimately it doesn’t change anything afterwards. […] You have to find another way to give that closeness the baby needs*.”

#### 3.3.2. Consequences for Parental Partnership

While one mother (M14) reported increased conflicts and lack of understanding for the situation by her partner, the majority emphasized the support they received from their partners (M01, M02, M03, M04, M05, M07, M08, M09, M10, M11, M13, M15). Although men may not have the best expertise in terms of breastfeeding as some mothers mentioned (M01, M03), the partners provided comfort and supported the women through their behavior and decisions (M01, M03, M05, M07, M08, M09, M10, M11, M13, M15), e.g., regarding the cessation of breastfeeding due to lack of success (M03: *“[…] because he said that no matter what decision I make now, he will support me in everything. And it will never be questioned whether I should have tried any longer”*). The mothers also reported that their partners also suffered from the situation when breastfeeding did not work out (M01, M04, M05, M07) and were, in part, relieved by the decision to stop breastfeeding (M05, M07).

#### 3.3.3. Social Effects, Responses, and Consequences

Some of the women (M06, M08, M15) reported social distancing due to conflicts (M06: “*And then she [her cousin] attacked me verbally via [Messenger app]. Why am I not breastfeeding and this is the best for my child […]. Since that, we do not have a good relation, since that point in time*”), due to the wish for some quiet time (M08: *“[…] because I didn’t desire to see visitors. […] everyone wants to come to visit and look at the baby, preferably every second day […] and I said: “No, I don’t want to have anyone here right now. And I have to find my way around first […]*””), and due to the baby himself/herself (M15: “*[…] he screamed a lot which has resulted in a little withdrawal from everyone.*”).

Other mothers were confronted with the lack of understanding for pumping breast milk (M02), or the whole situation (M05, M10). M05 reported: *“[…] I heard from all sides “Don’t act like that! […] Biting the bullet, pulling through, then it is good.” […] I didn’t get any encouragement from my best friend either. But it was always just “Yes, these are just bad excuses!” […]”*.

The situation also led to increasing conflicts for three mothers (M04, M05, M06). M04 told the interviewer that: *“every sentence, that was said at that time, was for me like provocation or evaluation”*. However, most of the women (also) experienced support, and reported positive reactions (M01, M03, M04, M05, M07, M09, M12, M14, M15). Some especially felt supported by their parents, and particularly by their own mothers (M05, M07, M09, M15).

#### 3.3.4. Experiencing Mothers Who Were Able to Breastfeed

One-third (M06, M08, M09, M10, M13) reported that they were delighted for every mother they saw breastfeeding their babies. M06 and M09 admired them for being able to breastfeed. However, eleven mothers (M01, M02, M03, M04, M07, M08, M10, M11, M13, M14, M15) had (also) negative feelings when they saw women nursing their babies.

These negative feelings emerged especially during group experiences (e.g., baby massage courses) when they were *“the only one who sat there with the bottle”* (M01) while all others were breastfeeding. The mothers received *“pitying looks”* (M01), felt *“not full-valued”* (M03), and experienced the situation of having to handle feeding bottle, water, and formula as inconvenient. M04 cried a lot because of this and did not want to meet other mothers any longer.

Some mothers were jealous of nursing mothers (M02, M03, M08, M11, M13, M14, M15). M08 subsumed that: “*[…] you rarely see people breastfeeding somewhere, but occasionally uh (clears her throat), when I’m somehow in another hospital or something, then there are freshly delivered, happy mothers sitting there breastfeeding their child in the café and you think like this, “Ooh, that could have been me.*””. Besides that, self-doubt up to self-blame was reported by the mothers in situations when confronted with nursing mothers (M01, M03, M04, M05).

#### 3.3.5. Feelings after Deciding to Stop Breastfeeding/to Express Breast Milk

Four mothers retrospectively regretted having stopped breastfeeding or expressing milk for their babies (M06, M08, M12, M15). These regrets were sometimes accompanied with feelings of bad conscience (e.g., M06: *“But now I would have liked to breastfeed. Thought if I had tried right, I would have stayed tuned”*, or M15: *“And then you have a very bad conscience to take away what nature has provided for the baby […] And that doesn’t even go away.”*). However, the decision had also been a relief for some of the mothers (M03, M04, M06, M08, M10, M12, M13, M14). They felt more relaxed (e.g., M03: *“So when I realized how relaxed everything was and how a load fell off”*), less stressed (e.g., M06: *“because you put yourself under a lot of stress, because you wanted it to work”*), and less pressured (e.g., M06: *“that the pressure is gone, that’s nice”*). Some mothers also described that they felt a discrepancy between relief and regret (M03, M05, M06, M08, M13). M13 looked at the decision *“with a laughing and a crying eye”*, describing mixed feelings. This was especially the case in specific situations, for instance, during courses with the baby (e.g., baby massage courses).

#### 3.3.6. Consequences for Potential Future Pregnancies

Thirteen women (M02, M03, M04, M05, M06, M07, M08, M09, M11, M12, M13, M14, M15) clearly enunciated that they would try to breastfeed potential future children. M07 said that *“everything is different with the second child”*. M11 reported that *“if it works out, it works out, then I will be extremely pleased. And when it doesn’t work out, then I know that they will grow up anyway.”*

M10 concluded not to try breastfeeding with a second child: “*[…] I don’t think I would want to breastfeed anymore. Because we drove so well with this bottle feeding. […] There is a selfish reason. Because [name of the baby] was only awake once the night. She drank her milliliters and then slept again. And then she slept alone in her crib in her room when she was five or six weeks old. And that’s an organizational question. It is easier. And I also think that closeness, mother and child, love can be given in other ways than through breastfeeding. […] So that’s why the decision would be—if the child didn’t come too early and needed a little more from mom […]: “Give me a tablet [for weaning].” Super.”*

M01 said that “*no additional children are planned (laughs), but if there would be a second child, I don’t think I would drive myself so crazy anymore. Or let me drive crazy by others*”. This idea was also reflected in the interviews with M02, M03, M04, M09, M11, M12, M13, M15, who all planned to be more relaxed regarding this topic with the (potential) second child.

### 3.4. Perceived Health Consequences for the Mother

In the following, the perceived consequences on physical (Section 3.4.1) and mental health (Section 3.4.2) of the mothers are described.

#### 3.4.1. Physical Health

Three mothers reported physical health problems due to the breastfeeding issue (M05, M14, M15). These were inflammation of the breasts (M05: *“[…] I had almost fever heat in my breasts […] because I had a starting inflammation in my breast due to galactostasis.”*), intense pain in the breasts (M14: *“It stings like a thousand knife sticks.”*), and weight loss (M15: *“[…] less weight than previous to pregnancy.”*).

#### 3.4.2. Mental Health

Thirteen mothers described psychological stress due to breastfeeding problems (M01, M02, M03, M04, M05, M07, M08, M09, M10, M11, M12, M14, M15). Most of them started experiencing psychological stress during the situation (e.g., M01: *“It was just too much for me. As a newly mother you […] are constantly being told: “You have to breastfeed your child now!” And it didn’t work and that was a big psychological burden for me.”*). Others noticed it only later *“how much it bothered me”* (M03). M08 mentioned that: *“[…] the child suffers from it insanely. At one point we both [i.e., baby and mother] just cried.”*. It was also reported that this psychological stress would be enhanced in specific situations. For instance, M01 told that she suffered a lot when the midwife wanted her to get an electric milk pump. M15 perceived shopping for baby formula as very stressful, because *“if you buy formula at [name of the drugstore], you will be approached by the shop assistants who will then tell you: “But you know, breastfeeding is best for your child!” These are slaps in the face.”*

The interviews also focused on postpartum depression (PPD). Ten mothers denied having had PPD (M02, M03, M04, M06, M08, M10, M11, M12, M13, M14). They described breastfeeding as an *“emotional topic”* (M03), indicated having had *“crying days”* (M02), and having cried for a longer time (e.g., M13: *“[…] I almost only cried in the hospital for two weeks.”*). Two other mothers (M01, M07) underlined that they felt mentally fragile, yet not to the extent of having PPD: *“So I wouldn’t say “depression”, it wasn’t that bad. But I was sad a lot […].”* (M07). Three mothers reported to having had PPD (M05, M09, M15), and described feelings of helplessness, aggression, sadness, and weakness, as well as mood changes. However, only one of these three mothers was diagnosed with PPD by a physician, took antidepressants, and received psychological assistance (M09).

## 4. Discussion

To the best of our knowledge, this study is among the first to cover the topic of breastfeeding problems and how young mothers perceive and manage them by using a qualitative approach. Our study shows that the inability to breastfeed can become a burden. The participants in our study tried to actively manage this situation by using nipple shields and breast pumps. It was found that the mothers would feel better when breast milk pumping succeeded because they could at least give their babies breast milk. However, when it failed, it further stressed the women. Feelings of failure and self-doubt, stress and pressure, guilt, disappointment, misery, and frustration were expressed in the interviews. These feelings were enhanced in specific situations, for instance, when they were confronted with mothers who were able to breastfeed. Some women reported that their social relationships had also suffered due to the situation. This could also include bonding with their babies, whereas stopping the attempt to breastfeed was perceived as a kind of a release from emotional pressure since many women felt more relaxed afterwards. Most women plan to be more relaxed regarding breastfeeding with the next baby and would therefore attempt to breastfeed again.

Previous quantitative studies have assessed the reasons for stopping breastfeeding [15,16,18,19]. However, the assessment was limited to pre-defined reasons due to the use of standardized questionnaires. Our qualitative approach has enabled us to make more detailed assessment of the reasons, and we were able to extend previous findings, although this aspect was only a secondary finding. This means that we were not only able to confirm quantitatively assessed reasons, such as the insufficient amount of breast milk, inflammation of the breasts, health problems, and issues related to the child [15,16] but also able to identify additional reasons. Future quantitative research on the reasons for weaning might extend the list of items based on the reasons identified in our study. In our study, reasons such as returning to work were not mentioned by the participants. However, such a reason might have very different consequences regarding the feelings that are associated with stopping breastfeeding early than the reasons that are less self-determined.

Involuntary ceasing of breastfeeding was found to be a very emotional topic for most of the women. The reported negative feelings are consistent with the findings of the few existing studies on this topic. In an Australian qualitative study, Ayton et al. investigated the experience of mothers who had to stop breastfeeding and found that it often led to “breastfeeding grief”, which is described as a prolonged sense of loss and failure [22]. The feelings of guilt and shame suffered by our participants were also found in a Norwegian sample [23]. In their study, the women indicated that they even felt that their decision in not breastfeeding was an act against the law. The feeling of guilt has not reached such a high level in our study, possibly since breastfeeding is cultivated more in Norway’s society compared to in Germany. Similar to our study, the women in the study of Lucchini Raies et al. [20] showed emotions such as distress and despair after recognizing the breastfeeding difficulties.

One major reason for feeling distressed and burdened was the unsuccessful use of the breast pump—an aspect which the majority of the mothers interviewed in our study were confronted with. We found that mothers’ success in using the breast pump, and thus their ability to at least give their own breast milk to their babies, was an important way of coping with the situation. It consoled these mothers to know that, at least to some extent, their children would benefit from their breast milk and its health advantages. Therefore, some of the mothers continued breast pumping for a very long time. We assume that successful use of breast pumps may help reduce the above-mentioned feelings of self-doubt and failure, while unsuccessful use may become an additional source of distress and pressure. This is also reflected in the work of Flaherman et al. [24], who found that breast pumping was associated with strong emotions, among them positive and negative ones. In their study, using the breast pump gave the mothers a sense of control, but afforded a lot of time and occasionally caused pain [24].

The demanding situation also led to behavioral changes when interacting with others. While close social relationships, including those with their partners, family members, and friends, predominantly supported the women, conflicts would arise with the more distantly related people. The existing literature mostly points out the supporting role of the child’s father in the early postpartum period [25,26,27]. In addition, we found that besides the fathers, communicating with someone who had experienced similar problems with breastfeeding would relieve some of the emotional burden. However, being in contact with and sharing of experience of mothers who were able to breastfeed could additionally stress the mothers in our sample, which was also concluded by Palmér [28]. Following her work, getting in touch with fellow female sufferers could be an important resource in handling this situation. In line with this earlier work, the results of our study suggest that it is important to give the affected mothers the opportunity to exchange feelings and experiences with other women having similar problems (e.g., in support groups or online forums). The midwives could create awareness among the women regarding these options.

Since it is common knowledge for young mothers and mothers-to-be that breastfeeding can have positive effects on mother–infant-attachment, some of our participants have frequently searched for other ways to foster closeness with their babies. This is also described as one of the possible reactions to breastfeeding problems in existing literature [28]. However, emotional or physical distance to the child, as reported by one-third of our mothers, should raise concerns. On the other hand, as shown by earlier research [29], secure infant attachment is not only associated with exclusive breastfeeding but also parenting. Therefore, mothers with breastfeeding problems should be informed about alternative parenting measures such as reading to the child and imparting maternal warmth and sensitivity (e.g., during bottle feeding), which can influence maternal bonding in a positive way [29]. This may enhance the well-being of mothers with breastfeeding difficulties.

After stopping breastfeeding, ambivalence was a common experience: Many women mourned, yet also felt relieved at the same time. Despite the fear of experiencing breastfeeding problems with the subsequent child, which is similar to the work of Palmér [28], nearly all participants in our study stated that they were planning to try breastfeeding again in the future, but with less pressure.

Pain, breast inflammation, and excessive weight loss were physical health problems that a number of mothers reported in this study. According to Abou-Dakn [1], 30% of all young mothers suffered from breast pain during breastfeeding, and 10–20% developed clinical signs of mastitis—thus, our results were in line with the existing data. However, mental health problems occurred even more frequently in our sample. Almost all women reported psychological stress due to the situation, and three mothers reported suffering from postpartum depression. However, only one mother in our sample was clinically diagnosed with postpartum depression. Nonetheless, providing clinical evidence on physical and mental health was not the aim of our qualitative study, since associations with breastfeeding have to be assessed and analyzed in large quantitative studies. Previous studies have already focused on the potential link between breastfeeding and stress in childbed [30], as well as breastfeeding and postpartum depression [1,21,31,32,33,34,35,36,37,38]. The direction of causality remains unclear. According to Krol and Grossmann [34], in gaining knowledge about the exact nature of breastfeeding problems, the concrete cessation reasons and the mood of the mother will be the essential themes in answering these questions. In getting a clearer understanding of these specific issues, further research which sets a particular focus on these topics is needed to adequately support women with breastfeeding problems.

There are some study limitations that should be considered in interpreting our findings. The aim of our study was to gain in-depth insight into the situation of mothers who experienced breastfeeding problems. However, since this qualitative study consisted of a convenience sample extended through snowballing, the generalizability of our findings may be limited. Although the situation may vary across individuals, we found a lot of overlapping and common themes between the interviews, which showed that this is a very emotional topic for many mothers who participated in our study. However, the “real” situation might have been worse than the one reflected in the interviews. There are at least two reasons that might lead to an underestimation: (1) since the interviews were conducted at least six months after giving birth, they might perceive the situation as less emotional retrospectively (recall bias); (2) women who perceived the situation as very dramatic and emotional might have been more likely to refuse participation in this study (participation bias). Nonetheless, our study provides valuable insights into the situation of mothers who want to breastfeed their newborn children yet face breastfeeding problems.

Future studies may use our findings for developing questionnaires and item sets for quantitative assessment. This would help to generalize our findings to the whole population in Germany and to other countries. Being aware of the aspects that women who experience breastfeeding problems are confronted with and a reflection on how they dealt with their situation may also be helpful for future campaigns that aim to strengthen breastfeeding. Although breastfeeding is the best option/choice for babies and must be further promoted, yet the fact that many women have been unsuccessful in breastfeeding and suffer due to this situation should be more highlighted in written information on breastfeeding. Besides that, our findings may also sensitize health professionals working with new mothers when they encounter breastfeeding problems.

## 5. Conclusions

Our findings show that the inability to breastfeed can have severe consequences on the mental and physical health of a mother, as well as on the mother’s social relationship, including her relationship with the baby. These mothers are in a dilemma: they want to breastfeed and to follow the recommendation of WHO and other health organizations, yet they are unable to. Based on this, they experience various emotional, cognitive, and social consequences.

Our findings underline that this specific group of mothers needs to be treated in a sensitive way by health professionals. Their needs may differ from the needs of mothers who do not want to breastfeed and those who want to breastfeed and do not encounter any problems. Based on our findings, this specific group seeks understanding of their situation, for emotional support, and for time for conversation. Introducing a breast pump in a sensitive way may help women to have a good feeling because they are “at least” able to provide their babies with breast milk. Furthermore, helping in establishing contact with mothers with similar problems could help overcome negative feelings such as self-doubt and failure. All in all, talking to these women in an understanding way without putting them under pressure may help that they do not give up their breastfeeding attempts too early. In addition, a more comprehensive preparation during prenatal classes about potential breastfeeding problems, potential problem solving (such as correct latching), use of formula, and the knowledge that unsuccessful breastfeeding is not automatically associated with negative consequences for the mother–infant-bonding may take the pressure off the women. Our findings need to be investigated in more detail in future studies, including different geographical regions.

## Figures and Tables

**Table 1:** Characteristics of the participating mothers.

ID	Age of Mother [Years]	Number of Children	Children with Breastfeeding Problems	Age of Firstborn Child (Months)	Age of Second-Born Child (Months)	Age of Mother at First Child’s Birth (Years)	Highest Vocational Qualification
M01	34	1	1	25		31	doctorate
M02	32	2	1	48	22	28	completed vocational training
M03	37	1	1	37		34	university degree
M04	28	1	1	28		26	University degree
M05	25	2	2	76	25	18	none
M06	34	3	3	39	12 *	31	completed vocational training
M07	22	1	1	9		21	completed vocational training
M08	29	1	1	16		27	completed vocational training
M09	35	1	1	29		33	university degree
M10	36	1	1	42		33	completed vocational training
M11	28	1	1	25		26	completed vocational training
M12	41	1	1	66		35	completed vocational training
M13	33	1	1	14		32	university degree
M14	40	2	1	33	11	37	university degree
M15	35	1	1	6		34	completed vocational training

All participating women reported problems in breastfeeding for at least one of their children; * twins

**Table 2:** Mean themes and subthemes extracted from the interviews.

Main Themes	Subthemes
Individual situation regarding breastfeeding (see Section 3.1)	Duration of breastfeeding (see Section 3.1) Presumed main reasons for being unable to breastfeed or having problems in breastfeeding (see Section 3.1)
Managing the situation (see Section 3.2)	Active managing regarding feeding of the baby (see Section 3.2.1) Cognitive-emotional responses related to managing the situation (see Section 3.2.2)
Perceived consequences for relations, feelings, and potential future pregnancies (see Section 3.3)	Interaction with the child (see Section 3.3.1) Consequences for parental partnership (see Section 3.3.2) Social effects, responses, and consequences (see Section 3.3.3) Experiencing mothers who were able to breastfeed (see Section 3.3.4) Feelings after deciding to stop breastfeeding/to express breast milk (see Section 3.3.5) Consequences for potential future pregnancies (see Section 3.3.6)
Perceived health consequences for the mother (see Section 3.4)	Physical health (see Section 3.4.1) Mental health (see Section 3.4.2)

## Data Availability

Data are available from the corresponding author upon reasonable request.

## Supplementary Materials

The following are available online at www.mdpi.com/article/10.3390/healthcare9101352/s1, Table S1: Presumed main reasons for being unable to breastfeed or having problems in breastfeeding; Table S2: Managing the situation: Cognitive-emotional responses related to managing the situation.
