# Supplementary material for: “It Is a Very Emotional Topic for Me”—Managing Breastfeeding Problems among German Mothers: A Qualitative Approach
Source: Healthcare (Basel). 2021 Oct 11;9(10):1352. doi: 10.3390/healthcare9101352 (PMC8544576; doi:10.3390/healthcare9101352)
Supplement: Supplementary file 1 [file healthcare-09-01352-s001.zip › healthcare-1385364-supplementary.pdf]

Table S1. Presumed main reasons for being unable to breastfeed or having problems in breastfeeding.

Subtheme	Reported by	Examples of quotes
Baby itself	M01<br>M02<br>M07<br>M09<br>M10<br>M11<br>M12<br>M14<br>M15	M01: "…he was tired, too. That was a difficult birth, it took more than 26 hours, then the child was exhausted [...] because my son was also very small after the birth and (..) was very light and powerless."<br><br>M02: "My son has a cleft lip and palate [...]."<br><br>M07: "He was very sleepy at the beginning, you hardly got him awake, he has 14 hours slept through and you were worried and thought: "Oh God, he must be hungry.""<br><br>M09: "I really think it was because it was too tiring for her so often. So, as I said, she wasn't as strong at the beginning [...]. She just put on weight slowly, and that was just too tiring for her. So you really noticed that she fought there. [...] I just think that she didn't suck enough."<br><br>M11: "This suction reflex was not pronounced enough."<br><br>M12: "As I said, he [the son] tried, but he just had these sucking difficulties for four weeks [...]"<br><br>M14: "And actually, the child is supposed to look for the breast, but they gave the child the breast at that time and I believe that she just got used to it incorrectly, because she did not open the mouth properly [...]"
Early start of (additional) bottle-feeding	M02<br>M03<br>M05<br>M06<br>M07<br>M08<br>M13<br>M14	M03: "It came out of the bottle much faster, it was always readily available, even in the amount he wanted."<br><br>M07: "[...] and then at some point nipple shields, which then didn't work anymore, because then the bottle / then he got used to it and yes, it is of course easier to drink from the bottle than from the breast."<br><br>M08: "He [the son] didn't manage the jump between bottle and breast anymore. It was too much confusion for him. And so I had to decide against breastfeeding at some point, unfortunately."<br><br>M13: "Of course, I still tried it [i.e., breastfeeding] myself from time to time, but he just didn't drink properly. He was already used to the bottle, probably. Everything else is a bit more exhausting, I suppose."<br><br>M14: "[...] And I think they kind of gave her [the daughter] some kind of food from the bottle. And then she may have gotten used to it the wrong way from the beginning on [...]"
Insufficient amount of breast milk	M01<br>M03<br>M05<br>M06<br>M12	M01: "The little one was hungry, he roared with hunger and there wasn't enough breast milk."<br><br>M05: ""I can't [breastfeed]. Firstly, I no longer have milk, [...]""<br><br>M06: "[...] but it came nothing, nothing came out. [...] Milliliters only came. Ten to twelve with great difficulty, and that was too little. Of this, you cannot feed a child."

Mental condition of the mother	M04<br>M05<br>M06<br>M11<br>M12	I: “What would you say, what was the main reason that it didn't work out?”<br>M04: “My head.“<br><br>M05: “[...] Many people say it's just turning off your head and so on. But if the psyche doesn't go along with it, then you can't turn off your head.”<br><br>M06: “[...] and this stress and [...] it [i.e., breastfeeding] has to work and so on. This has led to the blockade instead of it starting to work.”<br><br>M11: “Because of the psychological pressure that you put on yourself, nothing [i.e., breast milk] came out.”
Problems with correct latching	M05<br>M07<br>M08<br>M14	I: “What would you say was the hardest thing back then? So overall, now in retrospect?”<br>M05: “With [name of the 1st child] the latching [...]”<br><br>M07: “I think the biggest problem was that I could never really latch him correctly. Because they just didn't have enough time to show me how it works properly.”
Painful breast	M05<br>M06<br>M08<br>M14	M05: “I was in pain and I could only hear from all sides: "Don't make such a fuss! They will pass again!"”<br><br>M08: “And the problem was at that point in time, my breasts / my nipples were already totally inflamed and completely damaged by sucking so that latching was totally painful.”<br><br>M14: “There were problems, I tried it [i.e., breastfeeding] for three months, almost three months, exactly, and then the nipples were so inflamed and they looked like they were about to fall off.”
Delivery through Caesarean section	M09<br>M13<br>M14<br>M15	M09: “[It was] a bit difficult that I had a caesarean section, an unplanned one, and then only after two hours or so did I really have my daughter with me [...] and then I actually expected [...] that I could directly latch her and there she reacted very reluctantly to the breast.”<br><br>M13: “[...] and then when we were at home, the midwife said to me: "Yes, it often does not work [i.e., breastfeeding] after a caesarean section," and she did not really help to try it at that time.”
Separation of mother and child after birth	M02<br>M06<br>M12<br>M13	M02: “Um, well, my son has a cleft lip and palate and, uh, was actually taken away from me immediately.”<br><br>M12: “[...] because I saw him for two minutes after giving birth, and then he was on the intensive child care unit and I was upstairs [on another unit].”
Potential genetic reasons	M01<br>M05<br>M13	M01: “So my mother says she couldn't breastfeed me either. I was bottle-fed from the beginning when I was little. Maybe it's just in the family. I don’t know. [...] but maybe it's just that it doesn't work for us.”<br><br>M13: “It can of course also be that it is exactly like my sister's and that it is simply not enough [i.e., breast milk]. No idea.”
Health problems of mother	M05<br>M06<br>M13	M05: “You have to squeeze the front of the breast so that the child can dock properly. But that was not possible with me.”

		M06: “What I have to mention is that I had breast surgery. They surgically removed a fibroadenoma on the right side. But I don't know whether that's the reason [for the problems in breastfeeding].”<br><br>M13: “I had a tumor on the placenta.”

Table S2. Managing the situation: Cognitive-emotional responses related to managing the situation

Subtheme	Reported by	Examples of quotes
Self-doubts and feelings of failure	M01<br>M02<br>M03<br>M04<br>M07<br>M09<br>M11<br>M13<br>M14<br>M15	M01: “It takes a lot out of you when you think: "Oh, I'm the biggest loser because I just can't breastfeed, yes! I can't give my child what everyone expects me to do [...]."”<br><br>M03: “The most difficult thing [...] was that it didn't fit into my worldview.”<br><br>M04: “[...] then you got comments so that you have no stamina or because you quickly reached for the bottle. Yes. It's depressing. So it's not a nice feeling.”<br><br>M07: “[...] but I also pretended, what if we lived in the Stone Age or in a Third World country? Would my child have starved to death or would I have gotten it somehow?”<br><br>M11: “Uh (.) well, so shortly after pregnancy you are a bit more sensitive anyway, yes, and uh, then I (.) then I cried a little, so I cried, and um, of course, I beat myself up a little bit. (laughs) "Why doesn't it work now? What did I do wrong?" [...] so you do have your self-doubt.”<br><br>M13: “Yes a little bit, such a / such a feeling of failure was then already there .But at some point you just have to become realistic.”<br><br>M15: “Because you really torture yourself, you would like that, but then it will not work and you will feel as if you have failed. So, like you can't do it. Everyone else can do it. You can't do it. And all the advertising and so on always tells you: "Breastfeeding is best for the child."”
Feeling burdened with additional amount of work	M02<br>M03<br>M05<br>M09<br>M10<br>M12<br>M13<br>M14<br>M15	M03: “[...] I still believe that it is definitely the best and most natural thing for the child, that it also has a lot of advantages in terms of, no idea, the practical things. You don't have to sterilize a bottle afterwards, you don't have to go out of the house and remember: "Do I have hot water, do I have cold water, do I have formula, bottles [...]?"”<br><br>M05: “[...] I think I have spent around 300, 400 € for [the baby] only on bottles, pacifiers and even more on milk formula. Sure, breast milk makes it easy! Yes, you attach your child, your body is constantly producing milk again, you have no dirt, you have no mud, you have no costs that come up to you.”<br><br>M09: “So we always had a huge bag full of things with us when we were traveling, even if we only went into town. [...] and that was pretty cumbersome.”

		M12: “And what may have annoyed me a bit / this pumping is of course, I admit, is a bit annoying and exhausting. (laughs) You imagine yourself putting on your child and it drinks by itself and instead you have to sit there and wait until this stupid, I say stupid, pump is finished at some point. Of course, that's not so nice [...] because you have to do it consistently in a certain rhythm. That means, theoretically, sitting down at home or even if you are somewhere out, actually take the thing with you. And that was at some point too (.) cumbersome for me.”<br><br>M13: “[...] of course, you have to get up at night, prepare this stupid bottle, whereas others may be able to stay in bed [...] and cuddle and breastfeed [...]”<br><br>M15: “[...] So I always had formula in the syringe and then put on this nipple shield and injected formula under the nipple shield, so that he [the son] could suck and get milk immediately, because it took so long until it [i.e. breast milk] shot off [...]”
Stress and pressure	M03<br>M06<br>M08<br>M09<br>M10<br>M11<br>M13<br>M15	M03: “[...] one was almost almost all day busy with feeding the child.”<br><br>M06: “[...] because you put yourself under a lot of stress because you wanted it to work out.”<br><br>M08: “[...] because I couldn't relax. I didn't have that relaxed attitude you always read about. "The mothers are in such a bubble for the first few weeks and everything is great and the baby is cute and sleeping is secondary." I didn't have that at all because I was under stress all day, all day. 24 hours. From morning to in the middle of the night and then the day started all over again.”<br><br>M10: “I rather had the feeling, I put myself under pressure and it was very difficult to endure this screaming child in front of the breasts.”<br><br>M13: “[...] So it was really a really stressful time [...]”
Guilt feelings	M05<br>M07<br>M08<br>M09<br>M11<br>M13<br>M15	M05: “I blamed myself, but at the moment I was just a nervous wreck.”<br><br>M07: “Well, I was really very sad and, yes, the guilty conscience was, I guess, the biggest problem.”<br><br>M09: “[...] Because, if you want to breastfeed, and it doesn't work, then you often have that, the bad conscience [...]”<br><br>M11: “But I just thought: "What kind of mother am I if I can't feed my child by myself?" So I really blamed myself somehow that maybe I might even be to blame [...]”<br><br>M15: “Very horrible. I was really sad that it just didn't work and, as I said, I felt totally guilty.”
Concerns about child	M03<br>M05<br>M07<br>M08	M03: “That means I always thought in my head that he couldn't get anything out of my breast.”

	M10<br>M13	M05: "The baby is hungry! It doesn't scream out of sheer fun, it is hungry. Because I couldn't calm her down by rocking, stroking, nothing, nothing at all. She was hungry."<br><br>M08: "I've always been more nervous because this baby just didn't drink."<br><br>M13: "[...] But in this situation, you are just afraid that the child isn't well [...]"
Disappointment and unaccomplished expectations	M01<br>M02<br>M03<br>M04<br>M11<br>M15	M02: "Yes, not being able to give my child the closeness that I had planned in advance by breastfeeding. […] that was not to the extent that I actually expected."<br><br>M04: "Yes, everything was somehow not as I had imagined."<br><br>M11: "It was very difficult because I really wanted it, like a normal birth that I did not get, I actually wanted at least that breastfeeding works. And if that doesn't work, it gets a bit annoying."<br><br>M15: "[...] so with me it was just more sadness that everything did not work as I had imagined [...]"
Mental overload	M03<br>M05<br>M06<br>M08<br>M11	M03: "[...] it is a very emotional topic for me. [...] And then she [the midwife] explained anything to me, what I have to buy now and how we proceed with boiling the bottles and all that stuff. And my husband listened to her and I was just standing on the stairs and crying because I can't manage to feed my child."<br><br>I: "How did you feel in this situation?"<br>M06: "I was a bit overwhelmed." (laughs)<br><br>M05: "No one has ever looked after me or my daughter [in the delivery room after birth]. [...] But in the end I was left alone. [...] And nobody helped me that my child was fed."
Misery	M01<br>M03<br>M07<br>M12<br>M15	M01: "I was devastated. I could not do it [i.e., breastfeeding the baby]."<br><br>M07: "I cried a lot. [...] But I was very sad, although one should be happy in the first time."<br><br>M15: "I was really sad, that it [i.e., breastfeeding] did not work out."
Frustration and rage	M06<br>M08<br>M09	M06: "[...] so I was annoyed that it [i.e., breastfeeding] didn't work."<br><br>M08: "I was extremely annoyed because somehow it [i.e., breastfeeding] didn't work at all."
Rationalizing	M03<br>M09<br>M14	M03: "Therefore, one always tried to whitewash the situation."<br><br>M14: "[...] I am a very pragmatic person and when it [i.e., breastfeeding] doesn't work, then it does not work."
